# Randomized Split-Mouth Study on Postoperative Effects of Palmitoylethanolamide for Impacted Lower Third Molar Surgery

**DOI:** 10.5402/2011/917350

**Published:** 2011-04-17

**Authors:** Christian Bacci, Giulia Cassetta, Bruno Emanuele, Mario Berengo

**Affiliations:** Department of Medical and Surgical Specialities and Unit of Oral Surgery, Institute of Clinical Dentistry, University of Padua, Via Venezia, 90, 35100 Padua, Italy

## Abstract

The aim of this study was to assess the efficacy of Normast 300 mg in reducing swelling and pain after the surgical extraction of impacted lower third molars. *Materials and Methods*. A randomized, split-mouth, single-blind study was conducted on 30 patients between 18 and 30 years of age requiring lower third molar extraction. Patients underwent bilateral extractions in a randomized sequence, one extraction being performed under Normast treatment. The Normast treatment involved 2 tablets a day for 15 days. The parameters assessed at each procedure were trismus, swelling, pain, NSAID consumption, postoperative complications, drug tolerability, and safety. The results obtained were processed using repeated measures analysis of variance. *Results*. Perceived postoperative pain was reportedly significantly milder on Normast treatment than control. The trend of the means differed over time (*P* < .0001) and between the two extraction groups (*P* < .0221). On the other hand, for edema and trismus, the trend differed over time for both groups but did not differ between the two groups. *Discussion*. Our analyses indicate that patients experienced significantly less postoperative pain when they were treated with Normast. *Conclusions*. Administering Normast improves the postoperative course—in terms of pain—after lower third molar extraction.

## 1. Introduction

The third molars, and those of the mandible in particular, are the most frequently impacted of all teeth, with an incidence ranging between 9.5% and 39%, depending on the source considered [1].

Impacted third molar extraction is therefore a common procedure in oral and maxillofacial surgery [2–10], but third molar surgery is associated with an incidence of intraoperative and postoperative complications in the range of 4.6% to 30.9% [4]. Patients complain of pain, swelling, and trismus being the factors that most negatively affect their quality of life [3, 11] in the postoperative period.

Most authors prescribe NSAIDs (nonsteroidal anti-inflammatory drugs) to prevent and treat postoperative pain and glucocorticoids to control edema and trismus [12].

The aim of the present study was to assess the efficacy of Normast 300 mg in reducing postoperative pain and swelling after surgery to extract impacted lower third molars. Normast was reported to the Italian Ministry of Public Health as a “food product for special medicinal purposes,” based on the European Community standards; the product has no known side effects and is generally well tolerated. The active ingredient is micronized palmitoylethanolamide (300 mg tablets).

Palmitoylethanolamide (PEA) has been proposed as a substance capable of modulating mast cell response to inflammation [13–15] and might represent a new approach to the treatment of inflammatory processes [16].

PEA is an endogenous endocannabinoid belonging to the N-ACETYL ETHANOLAMINES (NAE) superfamily [17, 18].

PEA is a naturally occurring substance isolated from soy lecithin, egg yolk, and ground peanuts; it is also contained in the lipid fraction of rat brain, liver, and skeletal muscle. The presence of PEA has been documented in the brain and bone marrow of mouse, in dog heart extracts, and in degraded human tissues and peritoneal macrophages. NAEs are also contained in the blood. There is proof of the presence of PEA, and other NAEs, in marine species as well as in mammals [18].

It has been suggested that PEA behaves like an endogenous ligand for the CB_2_ receptors [17, 18], a subgroup of endocannabinoid receptors that mediate analgesic effects, and their involvement in painful neuropathic and inflammatory states has been demonstrated [19]. The literature suggests that PEA only becomes effective as an analgesic in the presence of an inflammatory state [14], by downregulating mast cell activity in vivo, and thereby reducing tissue inflammation [13, 14, 17].

PEA is a compound with documented antinociceptive and anti-inflammatory effects [17, 18, 20], but it also inhibits food intake, reduces gastrointestinal motility, inhibits the proliferation of cancer cells, and has a neuroprotective action [17].

The effect of PEA on edema is both dose and time dependent [15]. 

## 2. Materials and Methods

A randomized, split-mouth, single-blind study was conducted on patients referred to the Department of Dentistry at the University of Padua. 

The study was conducted in accordance with the ethical principles for medical research on human beings established by the 1964 Helsinki protocol.

It was not necessary to obtain the approval of the local ethical committee because Normast is not registered as a drug but as a “food product for special medicinal purposes,” and it is already used in other medical specialties. 

### 2.1. Inclusion and Exclusion Criteria

Thirty patients were selected, aged between 18 and 30 years, who needed bilateral lower third molar extractions and who gave their informed consent to enrolment in the study. On medical examination, the third molars had to show no signs of inflammation or tooth decay. Each patient's X-rays had to show that the two lower third molars were identical in position, orientation, depth, and class of impaction according to the Pell and Gregory classification. 

Patients over 30 years old were ruled out, and so were patients needing monolateral extractions, individuals who had taken part in other clinical studies in the two months prior to enrolment for the present study, patients with systemic diseases and taking chronic pharmacological therapy, and cases thought unlikely to assure the necessary compliance. Patients with known allergies to NSAIDs were also excluded, as were those who could not take such medication for other health-related reasons (gastric disorders).

A preoperative clinical examination was conducted on the oral cavity, obtaining an orthopantomography and, where necessary, a CT processed using DentaScan [21].

Before proceeding, the whole procedure was explained to patients in detail and their informed consent was obtained. Patients were also given an explanatory letter for their general practitioner.

Each of the 30 patients was scheduled to undergo lower third molar extraction bilaterally, the two extractions being performed in two separate sessions approximately 30 days apart. The surgery was always conducted by the same qualified operator. In each individual, one extraction was completed while, on treatment with Normast, the other being considered as a control; the order of the two extractions was randomly assigned. For the treatment arm, patients had to take Normast at a dose of 2 tablets a day (one in the morning and one in the evening) for a period of 15 days (6 days before surgery and 9 days afterwards). 

### 2.2. Assessment Parameters

The following parameters were recorded:

- trismus: mouth opening range was assessed by measuring the distance between the incisors before surgery (*T*0) and on the 3rd and 7th days after surgery (*T*1 and *T*2) [3];
- swelling: facial edema was assessed by measuring the distances between the labial commissure and the tragus (following the trend of the cheek), and between the lateral canthus and the gonion, before surgery (*T*0) and on the 3rd and 7th days afterwards (*T*1 and *T*2, resp.) [3]; the measurements were obtained with a silk 2/0 suture thread, which was then placed against a ruler; to ensure reproducible measurements, the points between which the measurement was taken were marked with a felt tip pen;
- pain: the pain perceived by the patient was scored on a visual analog scale (VAS) 10 cm long, where 0 meant no pain and 10 meant the worst pain imaginable [3];
- NSAID consumption in the week after surgery;
- postoperative complications;
- drug tolerability and safety: recording any onset of adverse events at the end of the treatment arm.

On the day scheduled for surgery, in addition to collecting information concerning trismus, swelling, and pain perception at *T*0, an ad hoc report form was used to record the patient's gender and age, the tooth extracted (3.8 or 4.8), the class and depth of the tooth according to the Pell and Gregory classification, and its orientation according to the Winter classification, the duration of the procedure, the date of starting the Normast treatment, and its duration (for patients in the treatment arm).

For the data to record at *T*1 (on the 3rd day after surgery), that is, trismus, swelling, and perceived pain, patients were given an ad hoc report form and asked to record these measurements at home; patients received a telephone call to remind them to do so.

On the day of their operation, patients were also given a diary and asked to make an accurate note of any NSAIDs used during the postoperative period. 

On the 7th day after the procedure, patients returned to the outpatients clinic at the Department of Dentistry to have their stitches removed, when the measurements relating to *T*2 (trismus, swelling, and pain) were recorded as well as an assessment of healing, postoperative course, and any side effects of the Normast treatment. Patients also returned the previously distributed report forms and diaries on the use of analgesics. 

### 2.3. Statistical Analysis

The results were processed using repeated measures analysis of variance and were considered significant when *P* < .05. 

## 3. Results

Only 26 of the 30 patients enrolled completed the protocol, while 4 dropped out as a result of inadequate compliance: 2 forgot to take Normast for the full 15-day period; one failed to return for the second extraction; one only took Normast for 3 days, then abandoned the drug, reporting the “onset of palpitations lasting 2-3 hours approximately an hour after taking the tablets” (they were apparently taking no other drugs in combination with the Normast treatment).

The protocol also involved assessing NSAID intake in the first week postoperatively, but the results proved impossible to analyze statistically because the data were excessively heterogeneous: although patients had been given clear instructions on the type of drug to use, only 4 patients took the prescribed ibuprofen 600 mg for postoperative pain control; the other 22 spontaneously opted for alternative pain control drugs (paracetamol, nimesulide, and ketoprofen) and at highly variable doses.

There were 3 cases of alveolitis, two of which occurred in the same patient; this would seem to have been a patient-related complication rather than a drug-related issue. The findings for postoperative pain, mouth opening range, the distance between the lateral canthus and the gonion, and the distance between the labial commissure and the tragus were assessed by repeated measures analysis of variance using as explanatory variables age, gender, side of mouth, duration of the procedure, time since surgery, and the interaction between the treatment and the time since surgery. All the models were amply significant. 

The results showed that the means for postoperative pain, mouth opening range, and the distance between the labial commissure and the tragus changed significantly with time, irrespective of the Normast treatment or any of the other explanatory variables.

The “duration of the procedure” variable was never statistically significant. 

**Figure 1:**
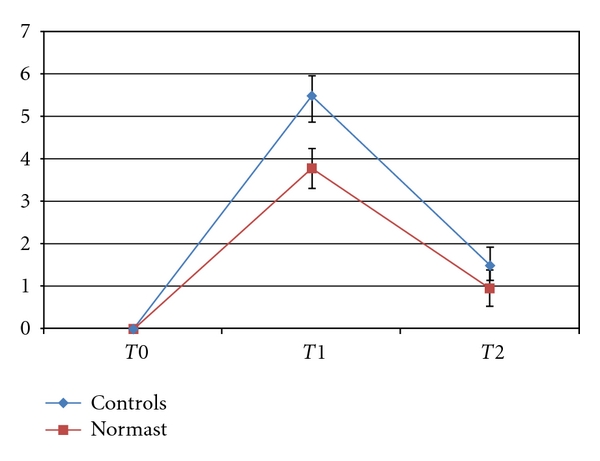
Trend of means ± standard errors for the VAS.

For postoperative pain control, the trend of the means differed over time (*P* < .0001) and also between the two extraction groups (*P* < .0221) (see Figure 1).

At *T*0, all the values recorded on the VAS were nil.

At *T*1, the mean VAS recorded by the patients taking Normast was 3.8 ± 3.09 cm, while in the control group it was 5.5 ± 2.42 cm; likewise, at *T*2, the mean VAS for the group on Normast treatment was lower than in the control group (1.0 ± 1.82 cm and 1.5 ± 2.18 cm, resp.).

**Figure 2:**
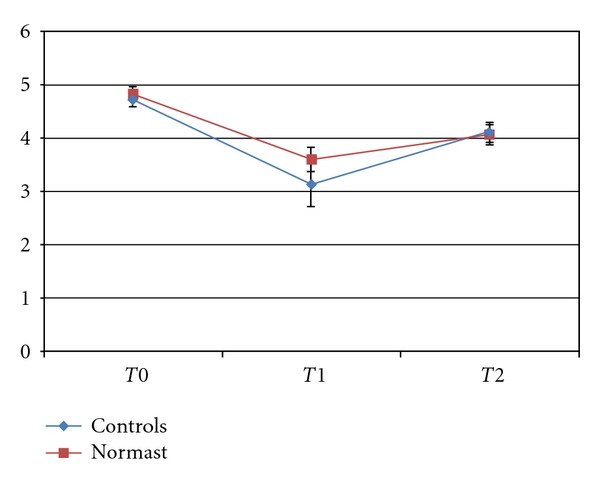
Trend of the means ± standard errors for the mouth opening range.

The trend of the means differed statistically over time for mouth opening range too, but it did not differ between the two extraction groups (see Figure 2); there was evidence of a significant difference between males and females however (*P* < .0241).

At *T*0, the mean values were 4.8 ± 0.59 cm and 4.7 ± 0.71 cm for the Normast-treated group and the control group, respectively.

At *T*1, the mean mouth opening range for patients on Normast treatment was 3.6 ± 2.21 cm, as opposed to 3.1 ± 1.18 cm for the control group.

At *T*2, the mean mouth opening range had increased to 4.1 ± 0.89 cm for the treated patients and 4.1 ± 0.93 cm for the controls.

None of the variables were significant for the distance between the lateral canthus and the gonion because of the marked variability of the measurements obtained.

At *T*0, the mean distance was 9.6 ± 0.91 cm and 9.6 ± 0.79 cm for the Normast-treated and control groups, respectively.

**Figure 3:**
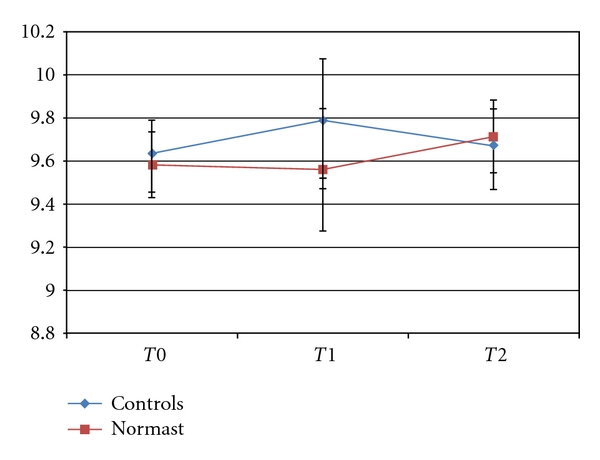
Trend of the means ± standard errors for the distance between the lateral canthus and the gonion.

At *T*1, the distance was 9.6 ± 1.63 cm in the treated group and 9.8 ± 1.46 cm in the control group, and, at *T*2, the figures were 9.7 ± 1.06 cm and 9.7 ± 0.86 cm, respectively (see Figure 3).

As for the distance between the labial commissure and the tragus, the trend of the mean measurements differed statistically over time (*P* < .0058), but not between the two treatment groups.

At *T*0, the mean distance for the group of patients treated with Normast was 10.2 ± 0.74 cm, while for the controls it was 9.9 ± 1.11 cm.

**Figure 4:**
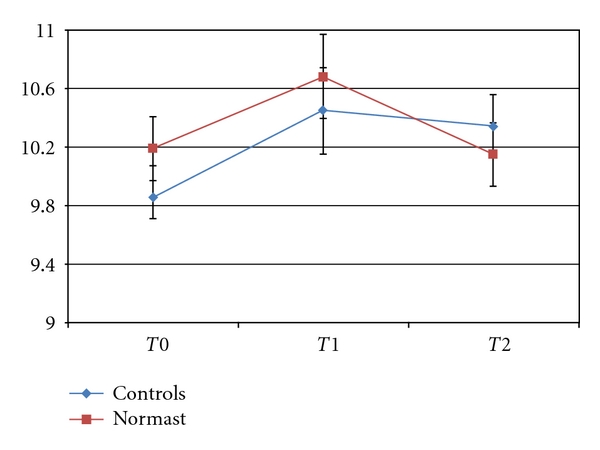
Trend of means ± standard errors for the distance between the labial commissure and the tragus.

At *T*1, the mean distance was 10.7 ± 1.53 cm for the Normast-treated group and 10.5 ± 1.46 cm for the controls, and, at *T*2, the figures were 10.2 ± 1.02 cm and 10.3 ± 1.11 cm, respectively (see Figure 4).

Two adverse reactions to Normast were reported by patients, that is, one case of drowsiness and one of palpitations.

## 4. Discussion

The statistical analysis produced statistically significant results inasmuch as concerns the correlation between the use of PEA and the perceived postoperative pain, as identified by means of a VAS.

As shown in Figure 1, while the baseline value was 0 at *T*0 for both groups, the mean score on the VAS at *T*1 and *T*2 was lower for the Normast treatment arm than for the control arm of the study.

Concerning trismus, the mean mouth opening range on the third postoperative day (*T*1) among the patients treated with Normast was 0.5 cm wider than that in the control group. Figure 1 shows, however, that the mean values for the two groups were comparable by postoperative day 7. This would suggest that Normast is able to improve the early postoperative course in terms of pain.

Two distances were considered to assess edema, that is, from the lateral canthus to the gonion and from the labial commissure to the tragus.

Figure 3 shows that the mean distances between the lateral canthus and the gonion were similar for the two groups at *T*0. Swelling was more severe in the control group than in the group treated with Normast at *T*1, then it gradually decreased up to day 7. The figure suggests that patients on Normast treatment suffered no edema from *T*0 to *T*1, but their swelling increased from the 3rd day onwards up until the 7th day, and at *T*2 the mean value was much the same as in the control group. As reported elsewhere in the literature [7, 22], this means that swelling in the control group increased up to the second or third postoperative day then regressed, disappearing completely between the 5th and 7th days after the extraction. The figure indicates that postoperative edema develops under treatment with Normast too, but its onset is delayed by comparison with the control group.

Figure 4 shows different mean values of distances between the labial commissure and the tragus for the Normast-treated and control groups at *T*0, with a virtually parallel rising trend up to *T*1, whereas, from *T*1 to *T*2, there is a faster reduction for the treatment group, which reaches lower mean values at *T*2 than in the control group.

Despite the impression given by the corresponding graphs, it is worth bearing in mind that the statistical analysis demonstrated that the trend for edema and trismus varied over time in both groups, but it did not differ between the groups.

As for the adverse events relating to the Normast treatment, one patient reported tachycardia developing about an hour after taking the tablets (this patient was ruled out of the study) and one other patient reported drowsiness after taking Normast. 

The patient with palpitations associated with the use of Normast reported taking no other drugs at the same time as the Normast treatment. About a week after being excluded from the study, the patient was asked to return to the outpatients clinic at the Department of Dentistry: when further details were obtained, the patient's medical history ultimately proved positive for the use of cannabinoids. There is nothing in the medical literature on any consequences of the joint use of natural or synthetic cannabinoids and PEA, but it may be that the side effects reported by this patient were the consequence of a synergic effect of PEA and tetrahydrocannabinol. 

## 5. Conclusions

According to the literature, patients complain of pain, swelling, and trismus as the main factors negatively influencing their quality of life [3, 11] after surgery for lower third molar extraction.

The postoperative course is commonly managed by prescribing NSAIDs to control postoperative pain and glucocorticoids for trismus and edema.

Our study demonstrated that combining Normast (palmitoylethanolamide) with the above-mentioned medication significantly reduces perceived pain, as recorded on a VAS, during the week after the surgical procedure.

For edema and trismus, on the other hand, our statistical analysis identified no significant differences between the extractions with and without associated Normast treatment.

We therefore conclude that, as concerns pain at least, treatment with Normast improves the postoperative course after impacted lower third molar extraction.
